# Supplementary figures and images for: SARS-CoV-2 Spike Protein Enhances Carboxypeptidase Activity of Angiotensin-Converting Enzyme 2
Source: Int J Mol Sci. 2024 Jun 6;25(11):6276. doi: 10.3390/ijms25116276 (PMC11172802; doi:10.3390/ijms25116276)

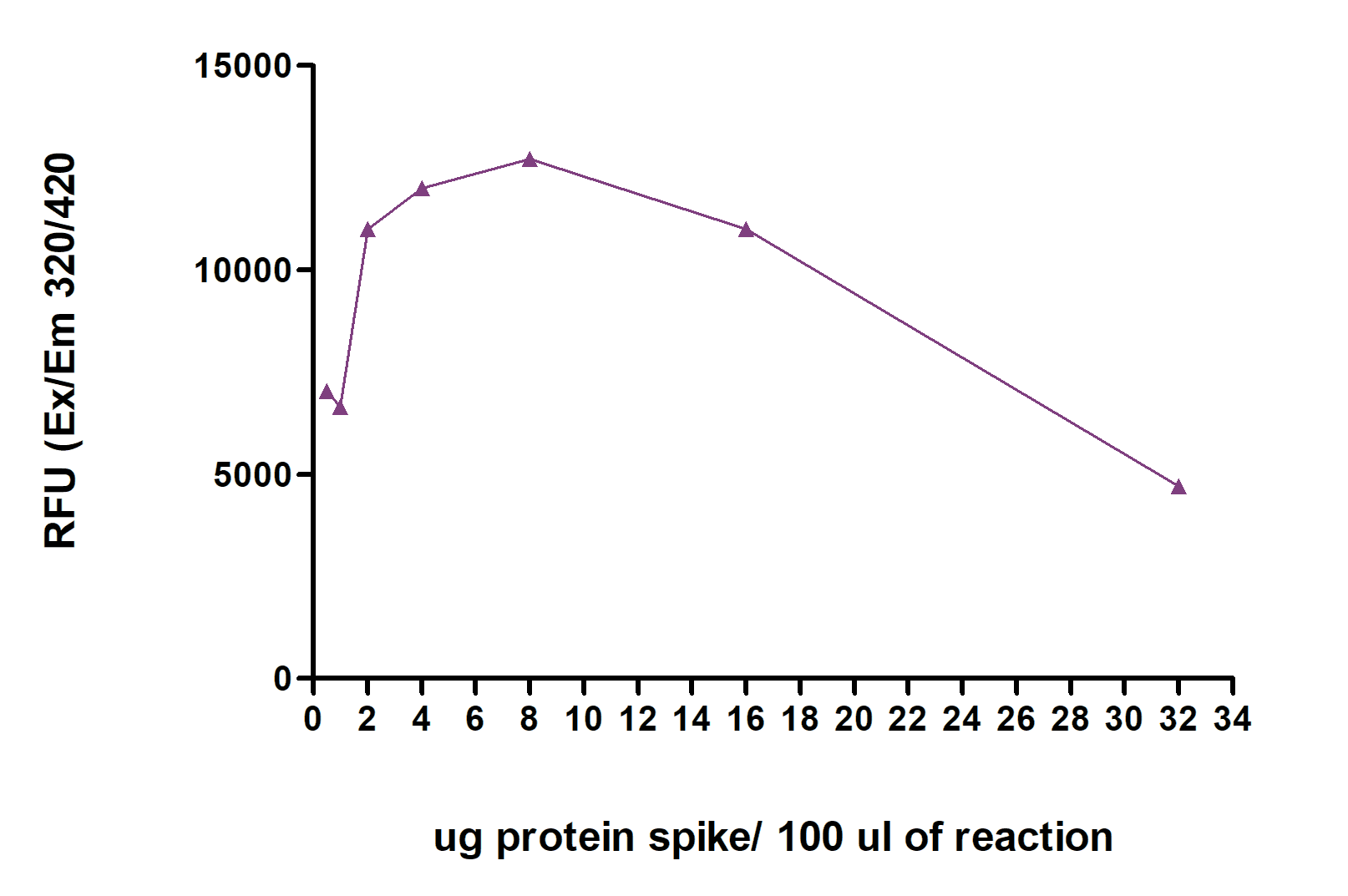

Supplement: Supplementary file 1 [file ijms-25-06276-s001.zip › ijms-3010920-supplementary.tif]
